# The relationship between person-centred care and well-being and satisfaction with care of patients living with obesity

**DOI:** 10.1093/intqhc/mzae078

**Published:** 2024-08-09

**Authors:** Paige I Crompvoets, Anna P Nieboer, Elisabeth F. C van Rossum, Jane M Cramm

**Affiliations:** Department of Socio-Medical Sciences, Erasmus School of Health Policy & Management, Erasmus University Rotterdam, 3000 DR Rotterdam P.O. Box 1738, The Netherlands; Department of Socio-Medical Sciences, Erasmus School of Health Policy & Management, Erasmus University Rotterdam, 3000 DR Rotterdam P.O. Box 1738, The Netherlands; Department of Internal Medicine, Division of Endocrinology, Erasmus MC, University Medical Center Rotterdam, 3000 CA Rotterdam P.O. Box 2040, The Netherlands; Obesity Center CGG, Erasmus MC, University Medical Center Rotterdam, 3000 CA Rotterdam P.O. Box 2040, The Netherlands; Department of Socio-Medical Sciences, Erasmus School of Health Policy & Management, Erasmus University Rotterdam, 3000 DR Rotterdam P.O. Box 1738, The Netherlands

**Keywords:** Person-centred care, patient experience, patient satisfaction, patient well-being

## Abstract

Person-centred care (PCC) is associated with improved patient well-being and higher levels of satisfaction with care but its impact on individuals living with obesity is not well-established. The main aim of this study was to assess the relationship between PCC and the physical and social well-being of patients living with obesity, as well as their satisfaction with care. This study is based on a cross-sectional, web-based survey administered among a representative panel of Dutch individuals living with obesity. The primary outcomes were physical and social well-being and satisfaction with care. The primary exposure was a rating of overall PCC, encompassing its eight dimensions. In addition, covariates considered in the analyses included sex, age, marital status, education level, body mass index, and chronic illness. The data from a total of 590 participants were analysed using descriptive statistics, correlation analyses, and multiple regression analyses. Among PCC dimensions, participants rated ‘access to care’ the highest (M 4.1, SD 0.6), while ‘coordination of care’ (M 3.5, SD 0.8) was rated lower than all other dimensions. Participants’ overall PCC ratings were positively correlated with their physical (*r *= 0.255, *P *< .001) and social well-being (*r *= 0.289, *P *< .001) and their satisfaction with care (*r *= 0.788, *P *< .001), as were the separate dimension scores. After controlling for sex, age, marital status, education level, body mass index, and chronic illness in the regression analyses, participants’ overall PCC ratings were positively related to their physical (*β* = 0.24, *P *< .001) and social well-being (*β* = 0.26, *P *< .001), and satisfaction with care (*β* = 0.79, *P *< .001). PCC holds promise for improved outcomes among patients living with obesity, both in terms of physical and social well-being, as well as satisfaction with care. This is an important finding, particularly when considering the profound physical, social, and psychological consequences associated with obesity. In addition to highlighting the potential benefits of PCC in the healthcare of individuals living with obesity, the findings offer valuable insights into strategies for further refining the provision of PCC to meet the specific needs of these patients.

## Introduction

The number of people living with obesity worldwide has nearly tripled since 1975 and continues to grow at a fast pace [1]. According to recent global estimates, obesity now affects more than a billion people worldwide [2]. Obesity is classified as a chronic, relapsing disease since it tends to persists over time, often requiring ongoing management due to the high probability of weight regain even after successful weight-loss attempts [3]. The development of obesity is usually a result of complex interactions among various genetic, behavioural, and environmental factors [4]. Obesity can have a strong impact on quality of life, with profound implications for the physical and social well-being of individuals [5]. These impacts are particularly notable for individuals living with more severe obesity and those managing multiple chronic conditions [6].

The physical consequences of obesity can be significant, giving rise to a wide range of issues that can cause discomfort and hinder participation in physical or social activities [7]. Some of the commonly reported physical problems are difficulties with mobility, chronic pain, respiratory issues, skin conditions, fatigue, and poor sleep quality [7–10]. Furthermore, obesity serves as a major risk factor for the development or worsening of other chronic health conditions, including cardiometabolic diseases, musculoskeletal disorders, some types of cancer, and mental disorders, which further implicate health and well-being [10, 11].

On top of physical challenges, many individuals living with obesity are subject to social stereotypes, prejudice, and unfair treatment because of their weight [12]. This phenomenon, known as weight stigma, seems to be most pervasive towards individuals living with severe obesity but it can affect anyone with excess body weight. Weight stigma is prevalent across many important life domains, such as personal relationships, education, employment, and healthcare [13]. Weight stigma can have detrimental effects on both physical and social well-being through various mechanisms, including increased exposure to stress, decreased quality and quantity of social relationships, compromised access to high-quality health care, and a decline in socio-economic status due to reduced opportunities and resources [14]. Moreover, perceiving weight stigma can trigger a weight-related social identity threat, causing individuals to become hyper-vigilant about potential rejection, resulting in social withdrawal, avoidance of health services, and other negative impacts on health and well-being [15].

Healthcare systems often fall short in effectively addressing the well-being concerns of patients living with obesity [16]. The current approach to care for these patients often revolves around tackling acute medical problems and recommending measures for weight reduction. This limited focus often results in short-term solutions that fail to address any underlying issues affecting patients’ well-being and hindering their weight loss efforts. As a result, patients commonly express dissatisfaction with their care, experiencing it as fragmented and ineffective, as their broader well-being concerns remain insufficiently addressed [17].

In an attempt to better meet the support needs of individuals with complex chronic conditions, many health systems are now moving towards a person-centred approach in which care is tailored to the specific preferences, goals, and circumstances of each individual. The Institute of Medicine defines person-centred care (PCC) as ‘care that is respectful of and responsive to individual patient preferences, needs and values; and ensuring that patient values guide all clinical decisions’ [18]. Extensive research identified eight broad dimensions of PCC that capture what is generally most important to patients: respect for patients’ preferences, physical comfort, the coordination of care, emotional support, access to care, the continuity of care, the provision of information and education, and the involvement of family and friends [19]. A review of PCC and its outcomes in 2013 clearly showed that organizations investing in these dimensions report more positive outcomes, such as greater patient well-being and satisfaction with care [20]. While the review included studies in various care settings and patient groups (e.g. diabetes care, cancer patients), it lacked studies within the context of obesity. To date, there remains a scarcity of data on PCC in obesity management, resulting in limited knowledge of its impact on patients living with obesity. While there are some articles on PCC for the management of obesity, they primarily focus on childhood obesity or are limited to case studies [21, 22]. Despite the anticipated benefits of PCC for patients living with obesity, the relationship between PCC’s eight dimensions and outcomes for this patient population remains unexplored.

This study aimed to address this knowledge gap by investigating the relationship between PCC and the physical and social well-being of patients living with obesity, as well as their satisfaction with care. Within a nationally representative sample, our objectives were to (i) explore participants’ experiences with PCC; (ii) determine bivariate associations of participants’ PCC experiences and background characteristics to their levels of physical well-being, social well-being, and satisfaction with care; and (iii) assess multivariate relationships between PCC experiences and participants’ levels of physical well-being, social well-being, and satisfaction with care, while controlling for background characteristics. We hypothesized that greater PCC would be positively related to all three primary outcome variables.

## Methods

### Study design

Our study was based on a cross-sectional, web-based survey administered by the Longitudinal Internet Studies for the Social sciences (LISS) panel (https://www.centerdata.nl/en/liss-panel). The panel is managed by Centerdata, an independent non-profit research institute affiliated with Tilburg University. The panel is based on true probability sample of households drawn from the Dutch population register by Statistics Netherlands. In 2022, the panel consisted of roughly 6500 individuals from about 4700 households. The panel members are compensated for participating in monthly web-based surveys, with necessary resources provided for households without a computer or internet access. The LISS panel abides by the European ‘General Data Protection Regulation (GDPR)’ and complies with all relevant ethical regulations. LISS participants give informed consent for the use of the collected data in scientific and policy-relevant research.

### Setting and participants

The target population of the study were individuals aged 18 years or older with obesity, defined as a body mass index (BMI) of at least 30 kg/m^2^. In July 2022, the survey was distributed among all panel members meeting these criteria (*n* = 896), yielding a total of 732 responses (82% response rate). BMI was based on participants’ most recent weight and height measurements, retrieved from a longitudinal survey fielded in November and December of each year. We verified any outliers in the data, resulting in the exclusion of five cases with incorrect BMI values. Given our interest in participants’ experiences with PCC, 130 cases who indicated ‘I do not know/not applicable’ to all PCC-related items were excluded. Finally, an analysis of survey completion times led to the exclusion of seven cases who completed the questionnaire faster than was deemed possible for meaningful responses. The final sample included 590 participants, which was considered sufficient to detect small to medium effects with a 95% confidence level and 80% power.

### Measures

To assess PCC, the survey included the 40-item person-centred obesity care instrument that assesses the eight dimensions of PCC (patient preferences, physical comfort, coordination of care, emotional support, access to care, continuity of care, information and education, and family and friends) among patients living with obesity [23]. The instrument is designed to be applicable across various care settings. Responses were given on a 5-point Likert scale from 1 (totally disagree) to 5 (totally agree). To minimize response bias, we allowed participants to select ‘I do not know/not applicable’ as well. Average dimension scores were calculated if ≥60% of the items were completed (all Cronbach’s *α* ≥0.87). Overall PCC ratings were calculated by averaging dimension scores for participants with at least five scores (Cronbach’s *α* = 0.92). Scores ranged from 1 to 5, with higher scores indicating better PCC.

The primary study outcomes were well-being and satisfaction with care. Well-being was assessed using the 15-item Social Production Function Instrument for the Level of Well-being, which measures both physical (comfort and stimulation) and social well-being (status, behavioural confirmation, and affection) [24]. Responses were given on a 4-point Likert scale from 1 (never) to 4 (always). Scores were averaged separately for physical (Cronbach’s *α* = 0.77) and social well-being (Cronbach’s *α* = 0.83), with higher scores (range 1–4) indicating greater well-being.

Satisfaction with care was assessed using a 6-item version [25] of the Satisfaction with Stroke Care questionnaire [26]. This scale was originally developed to evaluate satisfaction with inpatient care among stroke patients but has since been used to assess general satisfaction with care among various patient populations. Minor adjustments were made to the items (e.g. replacing ‘doctors’ with ‘healthcare professionals’). The resulting items were: ‘I have received all the information I want about the causes and nature of my health condition(s)’, ‘The healthcare professionals have done everything they can to improve my situation’, ‘I am satisfied with the type of care and support they have given me’, ‘I have had enough care and support’, ‘I am happy about the effects of the care and support on the progression of my condition(s)’, and ‘I am satisfied with the care and support that was provided’. Responses were given on a 4-point Likert scale from 1 (totally disagree) to 4 (totally agree) and scores were averaged across items (Cronbach’s *α* = 0.96), with higher scores (range 1–4) indicative of higher satisfaction with care.

In addition, we obtained information on participants’ socio-demographic profile (sex, age, marital status, education level) and BMI. Participants also reported on chronic illness using a validated inventory of 10 chronic conditions (e.g. type 2 diabetes or cardiovascular disease) and an option to disclose unlisted conditions [27].

### Data analysis

SPSS version 29 was used to perform the analyses. Dummy variables were created for marital status [living together with a partner (0), single (1)], education (low = primary or lower vocational, intermediate = secondary or intermediate vocational, high = higher vocational or university), and chronic illness [no chronic conditions (0), one or more chronic condition (1)]. Descriptive statistics included frequencies and percentages for categorical variables and mean and standard deviation for continuous measures. For continuous measures deviating from normality, the median and inter-quartile range is reported. To explore intragroup differences between PCC dimensions, a repeated measures ANOVA with Huynh–Feldt correction was performed, followed by Bonferroni-adjusted pair-wise comparisons. Bivariate associations among PCC and participants’ background characteristics, level of well-being, and satisfaction with care were identified using Pearson or Spearman correlation analysis, as appropriate. Correlations were classified as low (*r* ≈ 0.10–0.29), moderate (*r* ≈ 0.30–0.49), or high (*r* ≈ ≥ 0.50). To investigate multivariate relationships among PCC and participants’ physical and social well-being and satisfaction with care, while controlling for background variables, multiple regression analyses were performed. Assumptions of linear models (linearity, homoscedasticity, multicollinearity, multivariate normality, spurious outliers) were assessed and no large violations were observed. Statistical significance was set at two-sided 0.05, and Bonferroni-adjusted alpha levels are reported for multiple comparisons. An analysis of missing values (items with a >5% ‘not applicable’ response) revealed that participants without comorbid conditions had more missing data on some care-related items. In addition to the standard complete-case analysis (Supplementary Material 1), multiple imputations were used to estimate the overall association between PCC and participants’ physical and social well-being and satisfaction with care. The Markov Chain Monte Carlo algorithm was used to impute missing values 20 times with 50 iterations. Predictive mean matching was used as the imputation method.

## Results

Table 1 presents the descriptive statistics of the study sample. On a 1–5 scale, the mean overall PCC rating was 3.8 (SD 0.6). Participants rated ‘access to care’ (M 4.1, SD 0.6) the highest, followed by ‘patient preferences’ (M 4.0, SD 0.7), ‘physical comfort’ (M 3.9, SD 0.7), ‘continuity of care’ (M 3.8, SD 0.8), ‘information and education’ (M 3.8, SD 0.7), ‘family and friends’ (M 3.7, SD 0.8), ‘emotional support’ (M 3.7. SD 0.8), and ‘coordination of care’ (M 3.5, SD 0.8). A repeated measures ANOVA with Huynh–Feldt correction indicated significant differences in PCC scores across dimensions [*F*(5.662, 3334.781) = 97.473, *P* < .001]. Bonferroni-adjusted post-hoc comparisons revealed significant differences between most dimension scores, except those more closely aligned, such as patient preferences and physical comfort. Notably, participants rated ‘access to care’ significantly higher than all other dimensions, while ‘coordination of care’ was rated lower than all other dimensions (all *P* < .001). On a 1–4 scale, mean physical and social well-being scores were 2.6 (SD 0.5) and 2.7 (SD 0.5), respectively. Lastly, on a 1–4 scale, the mean satisfaction with care score was 3.0 (SD 0.6).

**Table 1.** Descriptive statistics of the study sample (*n* = 590).

Characteristic	Range	*n* (%) or mean (SD)
Sex (female)		337 (57.1%)
Age	18–92	59.22 (14.85)
Marital status (single)		202 (34.2%)
Education		
Low		196 (33.2%)
Intermediate		216 (36.6%)
High		178 (30.2%)
BMI^a^	30–59	33.37 (3.88); 32 (31–35)
Chronic illness (other than obesity)^b^		355 (60.2%)
Person-centred care^c^	1.8–5	3.83 (0.59)
Patient preferences^c^	1.6–5	4.02 (0.66)
Physical comfort^c^	1–5	3.94 (0.72)
Coordination of care^c^	1–5	3.48 (0.87)
Emotional support^c^	1–5	3.67 (0.84)
Access to care^c^	2–5	4.11 (0.55)
Continuity of care^c^	1–5	3.83 (0.75)
Information and education^c^	1–5	3.80 (0.67)
Family and friends^c^	1–5	3.71 (0.82)
Physical well-being^d^	1.3–4	2.63 (0.51)
Social well-being^d^	1.4–4	2.67 (0.47)
Satisfaction with care^d^	1–4	2.99 (0.58)

a Reported as mean (SD); median (interquartile range).

b Diabetes, cardiovascular diseases, heart failure, lung diseases, cancer, arthrosis, osteoporosis, chronic joint inflammation, depression, anxiety, or any unlisted chronic illness.

c Measured on a scale of 1–5.

d Measured on a scale of 1–4.

Participants’ overall PCC ratings correlated positively with their levels of physical and social well-being and their satisfaction with care (all *P* < .001). A low-to-moderate correlation was found between PCC and physical (*r *= 0.255) and social well-being (*r *= 0.289), whereas PCC and satisfaction with care were highly correlated (*r *= 0.788). Additionally, some of the background characteristics demonstrated low correlations with participants’ physical and social well-being, but not their satisfaction with care (Table 2; all *P* < .001). Participants’ age correlated positively with their physical (*r *= 0.145) and social well-being (*r *= 0.143), whereas single marital status correlated negatively with physical (*r *= −0.161) and social well-being (*r *= −0.170). BMI (*r *=−0.183) correlated negatively with participants’ physical well-being, as did the presence of one or more comorbid conditions (e.g. type 2 diabetes or cardiovascular disease; *r *= −0.204).

**Table 2.** Bivariate associations of patient characteristics and person-centred care to physical and social well-being and satisfaction with care among patients living with obesity.

	Physical well-being		Social well-being		Satisfaction with care	
Characteristic	*r*	*P*	*r*	*P*	*r*	*P*
Sex (female)	−0.080	.05	0.067	.11	−0.036	.40
Age	0.145	<.001*	0.143	<.001*	0.087	.04
Marital status (single)	−0.161	<.001*	−0.170	<.001*	0.007	.86
Education	0.038	.36	0.079	.06	−0.028	.52
BMI	−0.183	<.001*	−0.043	.30	−0.057	.19
Chronic illness (other than obesity)^a^	−0.204	<.001*	−0.075	.07	0.011	.82
Person-centred care	0.255	<.001*	0.289	<.001*	0.788	<.001*

* Significant at Bonferroni adjusted *α *= 0.007.

a Diabetes, cardiovascular diseases, heart failure, lung diseases, cancer, arthrosis, osteoporosis, chronic joint inflammation, depression, anxiety, or any unlisted chronic illness.

All PCC dimensions correlated significantly and positively with participants’ physical well-being, social well-being, and satisfaction with care (all *P* < .001; Table 3). Correlations with physical well-being and social well-being were relatively low in magnitude, while correlations with satisfaction with care were high.

**Table 3.** Bivariate associations of person-centred care dimensions to physical and social well-being and satisfaction with care among patients living with obesity.

	Physical well-being		Social well-being		Satisfaction with care	
Person-centred care dimensions	*r*	*P*	*r*	*P*	*r*	*P*
Patient preferences	0.207	<.001*	0.253	<.001*	0.628	<.001*
Physical comfort	0.184	<.001*	0.215	<.001*	0.526	<.001*
Coordination of care	0.229	<.001*	0.242	<.001*	0.656	<.001*
Emotional support	0.175	<.001*	0.228	<.001*	0.636	<.001*
Access to care	0.160	<.001*	0.203	<.001*	0.499	<.001*
Continuity of care	0.234	<.001*	0.230	<.001*	0.703	<.001*
Information and education	0.219	<.001*	0.248	<.001*	0.732	<.001*
Family and friends	0.167	<.001*	0.193	<.001*	0.502	<.001*

* Significant at Bonferroni adjusted *α* = 0.006.

The included covariates together explained 11% and 7% of the variance in participants’ physical (*R*^2^_adj_ = 0.11) and social well-being (*R*^2^_adj_ = 0.07), respectively (both *P* < .001). The covariates did not explain any of the variance in satisfaction with care. The addition of PCC in the models explained an additional 4% (*R*^2^_adj_ = 0.15), 7% (*R*^2^_adj_ = 0.14), and 62% (*R*^2^_adj_ = 0.62) of the variance in physical well-being, social well-being, and satisfaction with care, respectively (Table 4). In the adjusted models, PCC was positively related to all primary outcomes: physical well-being (*β* = 0.24), social well-being (*β* = 0.26), and satisfaction with care (*β*  = 0.79, all *P* < .001). Additionally, age (*β* = 0.14) and chronic illness (*β* = −0.21) were significant covariates for physical well-being, whereas age (*β* = 0.15) and single marital status (*β* = −0.16) were significant covariates for social well-being (all *P* < .001). Marital status and BMI showed significant associations with physical well-being in the bivariate analysis, but not in the adjusted multivariate analysis.

**Table 4.** Relationships of patient characteristics and person-centred care to physical and social well-being and satisfaction with care among patients living with obesity.

	Physical well-being		Social well-being		Satisfaction with care	
Variable	*β*(SE)	*P*	*β*(SE)	*P*	*β*(SE)	*P*
Sex (female)	−0.04 (0.04)	.32	0.10 (0.04)	.01	−0.04 (0.03)	.12
Age	0.14 (0.0)	<.001*	0.15 (0.0)	<.001*	0.0 (0.0)	.91
Marital status (single)	−0.12 (0.04)	.01	−0.16 (0.04)	<.001*	0.04 (0.04)	.11
Education^a^						
Low	−0.04 (0.05)	.42	−0.12 (0.05)	.01	0.02 (0.04)	.39
Intermediate	0.01 (0.05)	.76	−0.12 (0.04)	.01	0.02 (0.04)	.47
BMI	−0.10 (0.01)	.01	−0.01 (0.0)	.82	−0.02 (0.0)	.44
Chronic illness (other than obesity)^b^	−0.21 (0.04)	<.001*	−0.09 (0.04)	.03	0.0 (0.04)	.86
Person-centred care	0.24 (0.03)	<.001*	0.26 (0.03)	<.001*	0.79 (0.03)	<.001*
Adjusted *R*^2^	0.15^c^		0.14^d^		0.62^e^	

* Significant at Bonferroni adjusted *α* = 0.006.

a Reference group = high education.

b Diabetes, cardiovascular diseases, heart failure, lung diseases, cancer, arthrosis, osteoporosis, chronic joint inflammation, depression, anxiety, or any unlisted chronic illness.

c Adjusted *R*^2^ covariates = 0.11.

d Adjusted *R*^2^ covariates = 0.07.

e Adjusted *R*^2^ covariates = 0.00.

## Discussion

### Statement of principal findings

This study aimed to (i) explore the PCC experiences of patients living with obesity; (ii) determine bivariate associations of participants’ PCC experiences and background characteristics to their physical and social well-being and satisfaction with care; and (iii) assess multivariate relationships between participants’ PCC experiences and levels of physical and social well-being and satisfaction with care, while controlling for background characteristics. In a representative national sample, we found a high association of participants’ PCC experiences to their satisfaction with care, and low-to-moderate associations to their levels of physical and social well-being. In the adjusted multivariate analysis, we found positive relationships between PCC and all primary outcomes. This study thus showed that among patients living with obesity, experiencing greater PCC was related to increased satisfaction with care and greater physical and social well-being.

### Interpretation within the context of the wider literature

We found a stronger association between PCC and satisfaction with care compared to physical and social well-being. This difference is understandable when considering the nature of the different constructs. Previous research shows that satisfaction with care is primarily determined by health service characteristics [28]. While many studies have explored person-related factors in this context, the results have been inconclusive due to high variability in the findings. In our study, none of the background variables serviced as significant for patients’ satisfaction with care. In contrast, the physical and social well-being of individuals is shaped by a broad range of factors [24]. It is therefore not surprising that we found several links between patients’ background variables—such as age, marital status, and chronic illness—and their well-being outcomes. Interestingly, even after accounting for these variables, we still found a positive relationship between PCC and both physical and social well-being, suggesting that PCC may be an effective strategy for improving these patients’ well-being outcomes. This is an important finding, given the profound physical, social, and psychological implications of obesity, which can vary greatly among individuals.

Participants rated coordination of care lower compared to other PCC dimensions. Effectively addressing obesity poses certain challenges due to its multifactorial nature and the broad range of clinical presentations and associated comorbidities. This lower rating may reflect the challenges and shortcomings in the integration and organization of care services, which are frequently reported by patients living with obesity [29]. As a consequence of poor coordination, patients may experience fragmented care, where healthcare professionals from different disciplines involved in the care delivery struggle to communicate and collaborate effectively. This, in turn, can lead to critical issues such as missed information, misdiagnoses, and misunderstandings about the patients’ needs and preferences. Furthermore, our findings suggest that there may be room for improvement in other dimensions of care, such as the provision of emotional support and the involvement of family and friends. Current best practice in treating obesity prioritize long-term, sustainable changes, in which addressing psychosocial factors is considered a critical component [11]. Finally, participants in our study rated access to care higher than other dimensions. This could indicate that in this setting, few barriers were experienced in terms of accessing healthcare services. This contrasts with a recent study in England, where access to care was particularly low among people living with overweight and obesity, highlighting the variability in healthcare experiences across different geographical areas [30]. Notably, both studies found a lower rating for emotional support, suggesting that this may be an overlooked aspect of obesity care, warranting greater attention from healthcare providers and policymakers.

### Strengths and limitations

There were several strengths and limitations. First, the cross-sectional design of this study does not permit the establishment of causal relationships, warranting further research to evaluate the outcomes of PCC for patients living with obesity. Dynamic relationships between PCC and patients’ well-being and satisfaction with care cannot be excluded. Second, the study reported an average BMI of 33.4 (SD 3.9) kg/m^2^, but lacked information regarding waist circumference, an important marker of the amount of abdominal fat mass. This mean BMI suggests that the majority of participants fell into the categories of first- or second-class obesity. While this distribution aligns with that of the broader population, ensuring greater applicability of our findings, it is important to note that many studies have demonstrated that the consequences of obesity are most significant for those living with the most severe forms of obesity. Therefore, further investigation into how PCC relates to patient outcomes within this specific subgroup could reveal valuable insights. Furthermore, since this study relied on self-reported data, there was potential for reporting bias. To mitigate this risk, several measures were implemented. Outliers in BMI, for example, were cross-referenced, and participants were given the option to answer ‘I do knot know/not applicable’ for certain items to enhance the data’s reliability. Despite these limitations, there is sparse data on PCC for patients living with obesity, and this study is the first to document the importance of the eight dimensions of PCC for these important patient outcomes.

### Implications for policy, practice, and research

By considering the diverse circumstances of each individual, PCC allows for a more comprehensive understanding of patients and their support needs. Our findings suggests that such an approach holds promise for more effective care and improved outcomes among patients living with obesity. However, further research is necessary to establish causal relationships and gain deeper insights into the benefits and potential mechanisms through which PCC can positively influence the well-being and care experiences of patients living with obesity.

The current study suggests that addressing issues that stand in the way of coordinating and integrating health services may be particularly beneficial for improving the care for patients living with obesity, as well as enhancing other aspects of PCC, such as the provision of emotional support. These insights could be used by healthcare professionals and policymakers aiming to improve obesity care.

## Conclusions

In a cross-sectional, web-based survey among individuals living with obesity, we demonstrate that PCC is associated positively with both physical and social well-being, as well as with satisfaction with care. These findings are important given the considerable impact of obesity on the well-being of those living with obesity. The results underscore the potential benefits of prioritizing person-centred approaches in the healthcare of individuals living with obesity and provide valuable insight for improving the delivery of PCC to this specific patient population.

## Supplementary Material

mzae078_Supp

## Data Availability

The data that support this research were acquired through the LISS (Longitudinal Internet Studies for the Social Sciences) panel (https://www.centerdata.nl/en/liss-panel). Currently, the data are not yet available but will be accessible upon request from the LISS Data Archive once the research project is finalized. Users must comply with the data usage policies outlined by the LISS Data Archive.
