# Supplementary material for: The relationship between person-centred care and well-being and satisfaction with care of patients living with obesity
Source: Int J Qual Health Care. 2024 Aug 9;36(3):mzae078. doi: 10.1093/intqhc/mzae078 (PMC11363957; doi:10.1093/intqhc/mzae078)
Supplement: mzae078_Supp [file mzae078_supp.zip › Supplementary material.docx]

Supplementary material

Table A1.

*Relationships of patient characteristics and person-centred care to physical and social well-being and satisfaction with care among patients living with obesity based on complete-case analysis*

|  | Physical well-being | | Social well-being | | Satisfaction with care | |
| --- | --- | --- | --- | --- | --- | --- |
| Variable | *β (SE)* | *P* | *β (SE)* | *P* | *β (SE)* | *P* |
| Sex (female) | -0.02 (0.05) | 0.74 | 0.19 (0.04) | 0.01 | -0.03 (0.04) | 0.30 |
| Age | 0.17 (0.0) | <0.001* | 0.17 (0.0) | <0.001* | 0.01 (0.0) | 0.68 |
| Marital status (single) | -0.13 (0.05) | 0.01 | -0.12 (0.04) | 0.01 | 0.05 (0.04) | 0.08 |
| Education^1^ | | | | | | |
| Low | -0.04 (0.06) | 0.50 | -0.13 (0.06) | 0.02 | 0.03 (0.05) | 0.36 |
| Intermediate | 0.03 (0.06) | 0.54 | -0.14 (0.05) | 0.01 | 0.03 (0.04) | 0.38 |
| BMI | -0.08 (0.01) | 0.09 | 0.01 (0.01) | 0.79 | 0.03 (0.0) | 0.35 |
| Chronic illness (other than obesity)^2^ | -0.25 (0.05) | <0.001* | -0.10 (0.05) | 0.03 | -0.03 (0.04) | 0.29 |
| Person-centred care | 0.21 (0.04) | <0.001* | 0.28 (0.03) | <0.001* | 0.81 (0.03) | <0.001* |
| Adjusted R^2^ | 0.16 | | 0.14 | | 0.66 | |

Abbreviations: BMI, body mass index.

*Significant at Bonferroni adjusted α = 0.006.

^1^Reference group = high education

^2^Diabetes, cardiovascular diseases, heart failure, lung diseases, cancer, arthrosis, osteoporosis, chronic joint inflammation, depression, anxiety, or any unlisted chronic illness.
